# PDRPS7 protects cardiac cells from hypoxia/reoxygenation injury through inactivation of JNKs

**DOI:** 10.1002/2211-5463.12822

**Published:** 2020-03-16

**Authors:** Yulian Duan, Siyuan Cheng, Liang Jia, Zhao Zhang, Leilei Chen

**Affiliations:** ^1^ College of Life Sciences Nanjing Normal University China; ^2^ Department of Cardiology the First Affiliated Hospital of Nanjing Medical University, Nanjing Medical University China

**Keywords:** cardiomyocytes, hypoxia/reoxygenation, JNKs, PDRPS7, peptides

## Abstract

Myocardial ischemia/reperfusion (I/R) injury is a major complication of reperfusion therapy in myocardial infarction. Ischemic myocardium produces a variety of peptides. We recently identified PDRPS7 as a novel peptide in cardiomyocytes that can be induced by hypoxia. However, the role of PDRPS7 is unknown. Here, we investigated the effects of PDRPS7 on hypoxia/reoxygenation (H/R)‐induced injury in rat cardiomyoblast H9c2 cells and NRCMs. We found that PDRPS7 improved cell survival and attenuated lactate dehydrogenase leakage following H/R in H9c2 cells and NRCMs. PDRPS7 also alleviated H/R‐induced pulsation reduction in NRCMs. Moreover, H/R‐induced cell apoptosis was decreased in the presence of PDRPS7. H/R‐induced reactive oxygen species generation was reduced by PDRPS7; in addition, PDRPS7 did not impact H_2_O_2_‐induced cell injury. Signaling analysis demonstrated that H/R increased the phosphorylation levels of JNKs, ERKs, and p38 mitogen‐activated protein kinases. However, PDRPS7 only attenuated H/R‐induced JNK phosphorylation, but not phosphorylation of ERKs and p38. PDRPS7 protected cardiomyocytes from apoptosis by inhibiting JNK phosphorylation and c‐Jun phosphorylation pathways, markedly upregulating anti‐apoptotic Bcl‐2 expression and inhibiting that of pro‐apoptotic Bax and cleaved caspase‐3. Importantly, pharmacological activation of JNKs diminished the protective effect of PDRPS7 in terms of cell survival against H/R stimulation. In summary, our study identified PDRPS7 as a novel cardioprotective peptide against H/R challenge and this action was mediated, at least in part, through inactivation of JNKs.

## Graphical abstract

Novel inducible peptide PDRPS7 protects cardiomyocytes from H/R injury by reducing intracellular reactive oxygen species content and inhibiting the activation of JNKs and c‐Jun, specifically altering the expression of related molecules in the downstream apoptotic signaling pathway, including marked upregulation of anti‐apoptotic Bcl‐2 expression and inhibition of pro‐apoptotic Bax and cleaved caspase‐3.

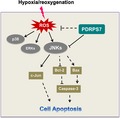

**Abbreviations**
Aniso: anisomycin
Bax: Bcl‐2‐associated X
Bcl‐2: B‐cell lymphoma‐2
CCPs: cell‐penetrating peptides
DCFH‐DA: 2′,7′‐dichlorofluorescein diacetate
ERKs: extracellular signal‐regulated kinases
H/R: hypoxia/reoxygenation
I/R: ischemia/reperfusion
JNKs: c‐Jun N‐terminal kinases
LDH: lactate dehydrogenase
MAPKs: mitogen‐activated protein kinases
MTT: 3‐(4, 5‐dimethylthiazol‐2‐yl)‐2, 5‐diphenyltetrazolium bromide
NRCMs: primary neonatal rat cardiomyocytes
p38: p38 mitogen‐activated protein kinases
ROS: reactive oxygen species

The incidence of myocardial ischemia is rapidly increasing around the world, and timely restoration of blood flow (reperfusion) to the ischemic myocardium is the standard treatment for patients. Reperfusion is demonstrated to limit infarct size, improve long‐term myocardial function, and, more importantly, reduce mortality. However, reperfusion can initiate a cascade of events that accelerate and extend postischemic injury, known as ischemia/reperfusion (I/R) injury 1, 2. Therefore, management of myocardial I/R injury is important for improving the outcome of these patients.

The pathogenesis of myocardial I/R injury includes multiple complex processes involving numerous signaling pathways and molecular players and ultimately results in cardiomyocyte death and cardiac dysfunction. Among them, the family of mitogen‐activated protein kinases (MAPKs) has been shown to play critical roles in the development of myocardial I/R injury 3, 4, 5. There are three major members of the mammalian MAPK family based on their preferential activation by extracellular stimuli: c‐Jun N‐terminal kinases (JNKs), p38 MAPKs (p38), and extracellular signal‐regulated kinases (ERKs) 6. Activation of JNKs, p38, and ERKs has been observed in experimental I/R myocardium. ERK activation has been shown to protect the heart against I/R injury by modulating pro‐apoptotic and prosurvival signaling 7, 8. However, numerous studies have demonstrated that JNKs‐ and p38‐mediated signaling pathways play an essential role in myocardial I/R injury 9, 10, 11. Indeed, p38 and JNK inhibition using either genetic modulation or chemical inhibitors protects cardiomyocytes against I/R injury 11, 12. Therefore, inhibitors of p38 and JNK activation serve as promising therapeutic potentials for myocardial I/R injury.

The I/R myocardium produces a variety of substances such as endogenous active peptides 13, 14, 15. As examples, intermedin, a member of the calcitonin gene‐related peptide superfamily, shows cardioprotective effects against I/R injury in rat and H_2_O_2_‐induced injury in primary cardiomyocytes through activation of ERKs 16. Moreover, other peptides such as adrenomedullin, bradykinin, ANP, BNP, CNP, and urocortins are all markedly protective against experimental myocardial I/R injury through a series of cytoprotective signaling pathways during early reperfusion 17. Of particular interest, we have recently identified 220 differentially expressed peptides originating from 119 proteins in cardiomyocytes that were exposed to hypoxia, of which 37 differentially expressed peptides showed upregulated expression 18. However, the biological roles of these identified peptides are unknown. In this study, we selected the polypeptide PDRPS7, one of the 37 upregulated expressed peptides, and found it exert cardiac protection against hypoxia/reoxygenation (H/R) injury through inactivation of JNKs.

## Reagents and methods

### Chemicals and antibodies

2′,7′‐dichlorofluorescein diacetate (DCFH‐DA) was purchased from Sigma‐Aldrich (St Louis, MO, USA). Hoechst 33342 reagent, Dulbecco's modified Eagle's medium (DMEM), and FBS were obtained from Invitrogen (Carlsbad, CA, USA). Primary antibodies for total‐JNKs and phosphor‐JNKs, total‐ERK1/2 and phosphor‐ERK1/2, total‐p38 MAPK and phosphor‐p38 MAPK, total‐Akt and phosphor‐Akt, total‐c‐Jun and phosphor‐c‐Jun, B‐cell lymphoma‐2 (Bcl‐2), Bcl‐2‐associated X (Bax), cleaved caspase‐3 were obtained from Cell Signaling Technology (Beverly, MA, USA). Primary antibody against GAPDH was from Bioworld Technology (Bloomington, MN, USA). 3‐(4, 5‐dimethylthiazol‐2‐yl)‐2, 5‐diphenyltetrazolium bromide (MTT) reagent was from Bio Basic Inc (Markham, ON, Canada). Anisomycin (Aniso) was from MedChemExpress (New Jersey, MA, USA). Protease inhibitor cocktail was from Roche (Mannheim, Germany). BCA protein assay kit and SuperSignal West Pico chemiluminescent substrate were obtained from Pierce (Rockford, IL, USA). Lactate dehydrogenase (LDH) detection kit was from Jiancheng Bioengineering (Nanjing, China). TUNEL assay kit was from Beyotime Biotechnology (Nantong, China).

### PDRPS7 polypeptide synthesis

The PDRPS7 peptide sequence is TGKDVNFEFPEFQL, but the working sequence of PDRPS7 is Ac‐RKKRRQRRRA‐TGKDVNFEFPEFQL‐NH_2_ (Fig. 2A). Among them, the RKKRRQRRRA sequence was used as a cell‐penetrating peptide (CCP) that assists PDRPS7 cross the cell plasma membrane. Additional groups, Ac at the amino terminal and NH_2_ at carboxyl terminal, were used to maintain peptide stability. The peptide was produced by Shanghai Science Peptide Biological Technology (Shanghai, China) via solid‐phase synthesis.

### Cell culture and treatment

**Fig. 1:**
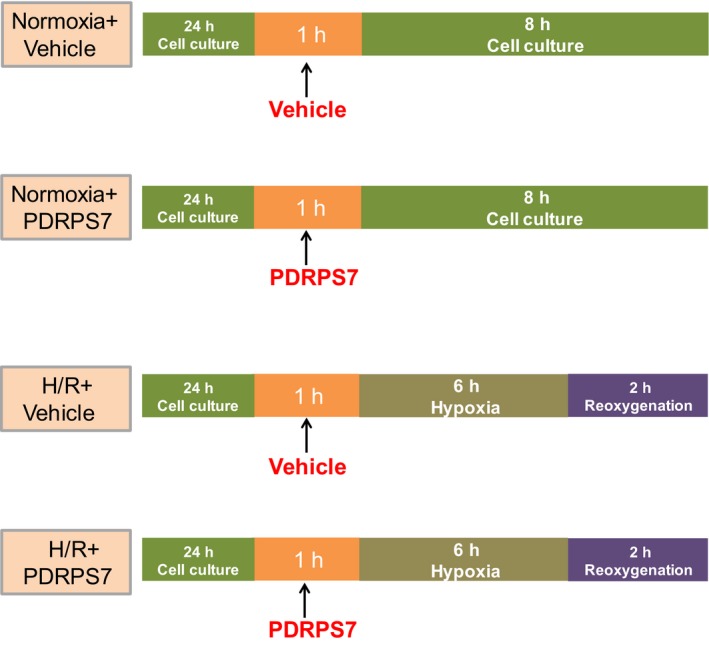
Experimental protocol. The schema shows the treatment schedules.

Rat cardiomyoblast H9c2 cells were obtained from ATCC (Manassas, VA, USA) and maintained in low glucose DMEM supplemented with 10% FBS. To induce hypoxia, cells were changed to serum‐ and glucose‐free DMEM and incubated in 94% N_2_, 5% CO_2_, and 1% O_2_ in a humidified tri‐gas incubator at 37 °C for 6 h. Subsequently, cells were reoxygenated by incubation with DMEM containing 10% FBS in 95% air and 5% CO_2_ at 37 °C for 2 h. For examining the effects of PDRPS7 on H/R‐induced cell injury, H9c2 cells were treated with PDRPS7 (50 μm) 1 hr prior to H/R. The sterilized ddH2O treatment with the same volume of peptides was used as the vehicle group. The treatment protocol is shown in Fig. 1.

In JNK activation experiments, H9c2 cells were pretreated with Aniso (250 nm) for 1 hr and then treated with PDRPS7 (50 μm) for 1 hr. Finally, cells entered the H/R treatment. The treatment protocol is shown in Fig. 10A.

### Acquisition of NRCMs and measurement of beating frequency

Primary neonatal rat cardiomyocytes (NRCM) cultures were prepared from 1‐ to 2‐day‐old Sprague–Dawley rat and incubated in DMEM media containing 10% horse serum (HS), 5% FBS, 1% penicillin/streptomycin at 37 °C in a 5% CO_2_ incubator. To induce hypoxia, NRCMs were changed to serum‐ and glucose‐free DMEM and incubated in 94% N_2_, 5% CO_2_, and 1% O_2_ in a humidified tri‐gas incubator at 37 °C for 6 h. Subsequently, NRCMs were reoxygenated by incubation with DMEM containing 10% HS, 5% FBS in 95% air, and 5% CO_2_ at 37 °C for 2 h. For examining the effects of PDRPS7 on H/R‐induced cell injury, NRCMs were treated with PDRPS7 (50 μm) 1 hr prior to H/R.

To analyze NRCMs beating frequency (beat/30 s), beating was recorded for 30 s at similar conditioned areas of syncytium by a camera and counted by a stopwatch online clock 19. The beating frequency of syncytium in each group was measured in four wells.

### Examination of cell viability and morphological changes

After treatments, cell viability was determined by MTT assay and LDH leakage, according to the manufacturer's instructions as described previously 20, 21. Cell morphology was examined using phase‐contrast light microscopy (Zeiss Ltd., Oberkochen, Germany).

### Apoptosis

H9c2 cells apoptosis was examined using a TUNEL assay kit as described in previous studies 22. Hoechst 33342 reagent was used to counterstain nuclei. The number of TUNEL‐positive cells was counted within more than eleven randomly chosen fields using a fluorescence microscope at a magnification of 200× (Olympus, Tokyo, Japan). The percentage of apoptotic cells over total cells was calculated.

### Measurement of intracellular ROS content

Intracellular reactive oxygen species (ROS) content was measured by DCFH‐DA assay as described in previous studies 20. After H/R treatment, DCFH‐DA (4 μm) was introduced to culture for 30 min. Hoechst 33342 reagent was used to counterstain nuclei. The staining was observed using a fluorescence microscope (400×) and quantified in eleven randomly selected areas of each sample using cellsens dimension 1.15 software (Olympus) 23.

### Western blot analysis

Western blot was performed as described previously 20, 22. Briefly, cytosolic fractions were extracted from H9c2 cells. Equal amounts of protein extracts were separated on 10% SDS/PAGE and transferred onto immobilon‐PVDF membrane (Millipore Corp, Bedford, MA, USA). After blocking, the membrane was incubated with primary antibody at 4 °C overnight, followed by incubation with the appropriate secondary antibody. The same membrane was also probed with anti‐GAPDH antibody for loading control. The blots were detected with an ECL kit, and signals were quantified by scanning densitometry.

### Statistical analysis

Results are expressed as means ± standard deviation (mean ± SD). Comparisons between groups were performed by Student two‐tailed unpaired *t*‐test, one‐way or two‐way analysis of variance (ANOVA) analysis followed by Tukey *post hoc* test. *P < *0.05 was considered to be significant.

## Results

### Characteristics of PDRPS7 polypeptide

**Fig. 2:**
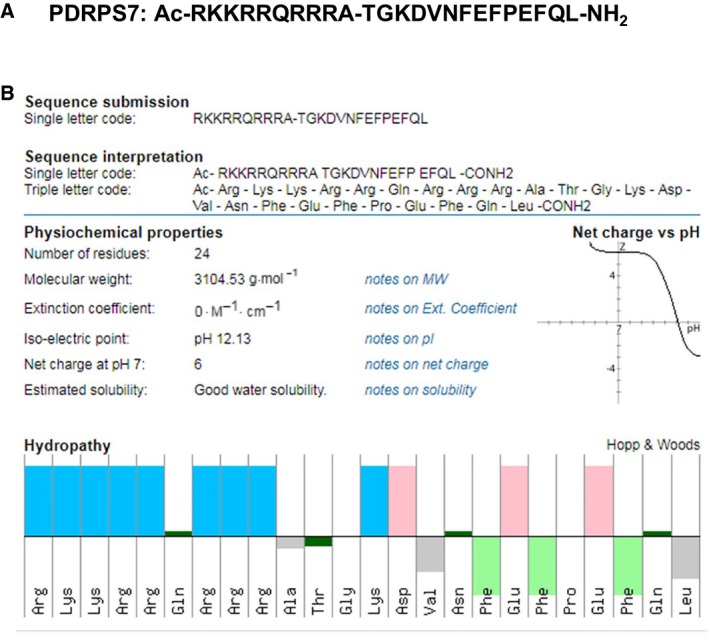
Characteristics of PDRPS7. The working sequence of PDRPS7 (A) and characteristics of PDRPS7 (B).

The characteristics of PDRPS7 polypeptide were obtained by inputting the polypeptide sequence at http://pepcalc.com/, which is a peptide property calculator. PDRPS7 contains 14 core amino acid residues and 10 CCPs. According to the property calculator, the molecular weight was 3104.53, isoelectric point: 12.13, net charge at a pH of 7: 6, and the estimated water solubility was good (Fig. 2B).

### PDRPS7 increases cell survival following H/R

**Fig. 3:**
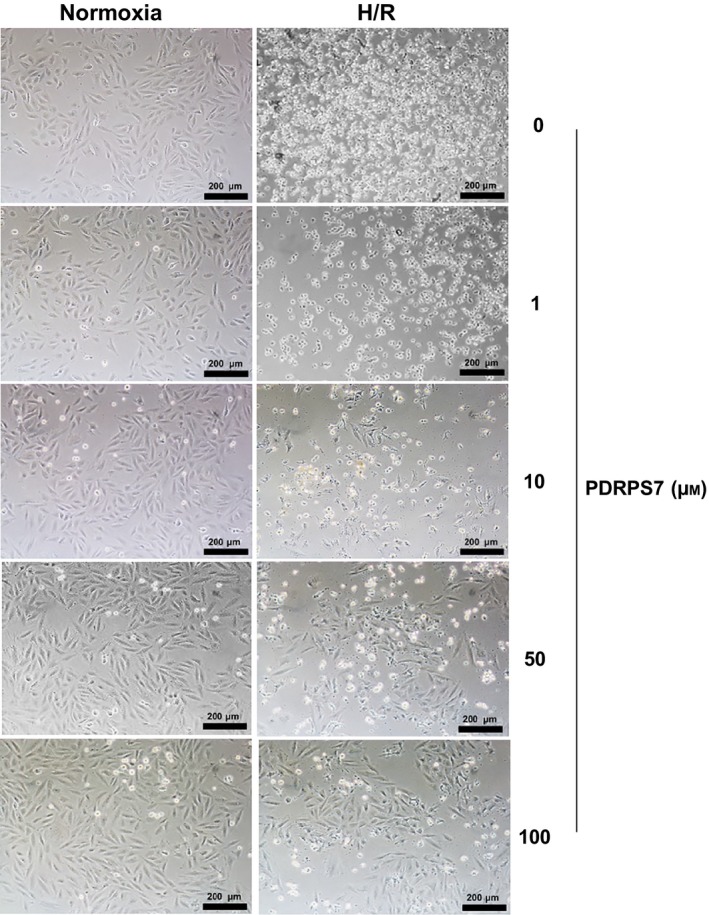
PDRPS7 attenuated cell morphological abnormalities following H/R. H9c2 cells were treated with PDRPS7 at the indicated concentrations 1 hr prior to H/R. Cell morphology was examined by phase‐contrast microscopy at a magnification of 100×. Scale bar = 200 μm. *n* = 3/group.

**Fig. 4:**
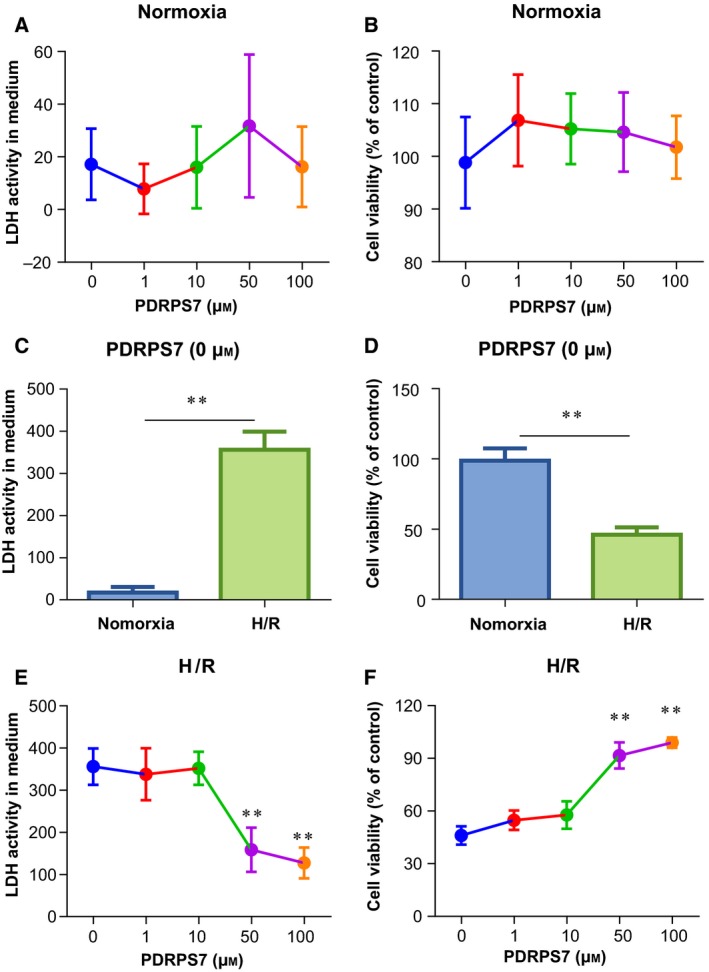
PDRPS7 improved cell survival following H/R. H9c2 cells were treated with PDRPS7 at the indicated concentrations 1 hr prior to H/R. Cell injury was determined by LDH leakage (A, C, E) and MTT analysis (B, D, F). **P < 0.01 vs. normoxia groups (C, D); **P < 0.01 vs. 0 μm PDRPS7 group (E, F). Data were shown as mean ± SD and analyzed using unpaired *t*‐test. *n* = 4/group.

To evaluate whether the peptide PDRPS7 plays a role in H/R‐induced injury in cardiac cells, we pretreated H9c2 cells with PDRPS7 at different concentrations. As Fig. 3 shows, we found the H9c2 cells were fusiform with a full cytoplasm and clear edges and PDRPS7 at different concentrations had no adverse effect on the cell state in the normoxia group. Differently, the H/R+ vehicle group showed cell morphological abnormalities, including applanate and shrunken shapes with loss of cellular integrity, were attenuated by PDRPS7 pretreatment at concentrations of 50 and 100 μm. Under normoxia conditions, PDRPS7 at concentrations of 0, 1, 10, 50, and 100 μm did not change cell damage as indicated by LDH leakage assay (Fig. 4A) and cell viability as indicated by MTT assay (Fig. 4B). We also noticed that H/R resulted in increased LDH activity in medium (Fig. 4C) and reduced cell viability (Fig. 4D) and, respectively, compared with that in their normoxia controls (*P < *0.01). Pretreatment with PDRPS7 at concentrations of 1 and 10 μm did not impact cell viability and LDH activities in culture medium of H/R‐treated H9c2 cells, respectively, compared with the H/R cells in the absence of PDRPS7 (Fig , 4E,F). However, 50 and 100 μm PDRPS7 pretreatment significantly improved cell viability and decreased medium LDH activity of H/R‐exposed H9c2 cells, respectively, compared with that in H/R cells in the absence of PDRPS7 (Fig. 4E,F, *P* < 0.01). Collectively, the data suggest the protective effects of PDRPS7 against H/R‐induced cell injury. Moreover, based on our observations, 50 μm of PDRPS7 was chosen for the subsequent experiments.

### Scrambled peptide shows no effect on H/R‐induced injury of H9c2 cells

**Fig. 5:**
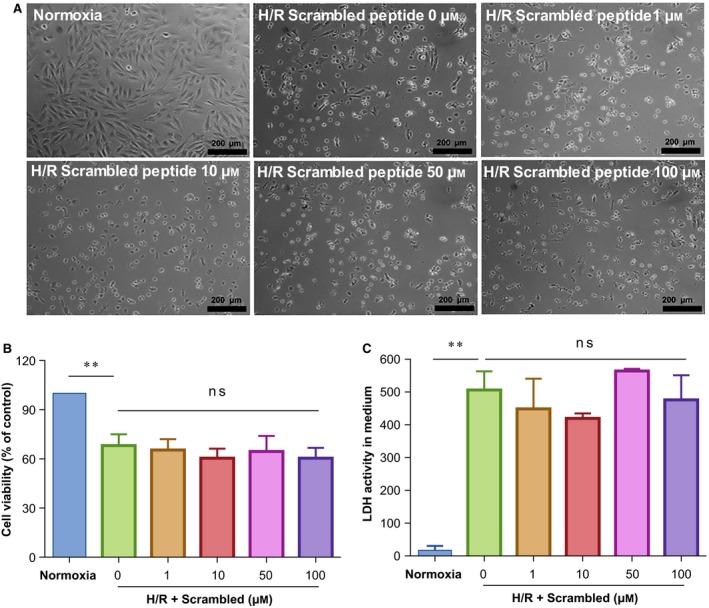
Scrambled peptide did not impact cell injury following H/R. H9c2 cells were treated with scrambled peptide at the indicated concentrations 1 hr prior to H/R. Cell morphology was examined by phase‐contrast microscopy at a magnification of 100×, scale bar = 200 μm (A), MTT analysis (B), and LDH leakage analysis (C). Data were shown as mean ± SD and analyzed using one‐way ANOVA followed by *post hoc* test. **P < 0.01 vs. normoxia groups; *n* = 4/group.

To confirm PDRPS7‐induced cell protection was attributable to the specific amino acid sequence, not only the amino acid species, we treated H9c2 cells with scrambled peptide 1 hr prior to H/R stimulation. We found that H/R‐induced cell morphological abnormalities were not obviously changed by the scrambled peptide at concentrations of 0, 1, 10, 50, and 100 μm (Fig. 5A). Moreover, MTT assay showed that H/R‐induced decrease in cell viability was not improved by the scrambled peptide at concentrations of 0, 1, 10, 50, and 100 μm (Fig. 5B). Additionally, LDH activity assay demonstrated that the H/R‐induced increase in LDH activity in culture medium was not reduced by the scrambled peptide pretreatment (Fig. 5C). Therefore, cell protective effects of PDRPS7 were attributable to its specific amino acid sequence.

### PDRPS7 decreases cell apoptosis following H/R

**Fig. 6:**
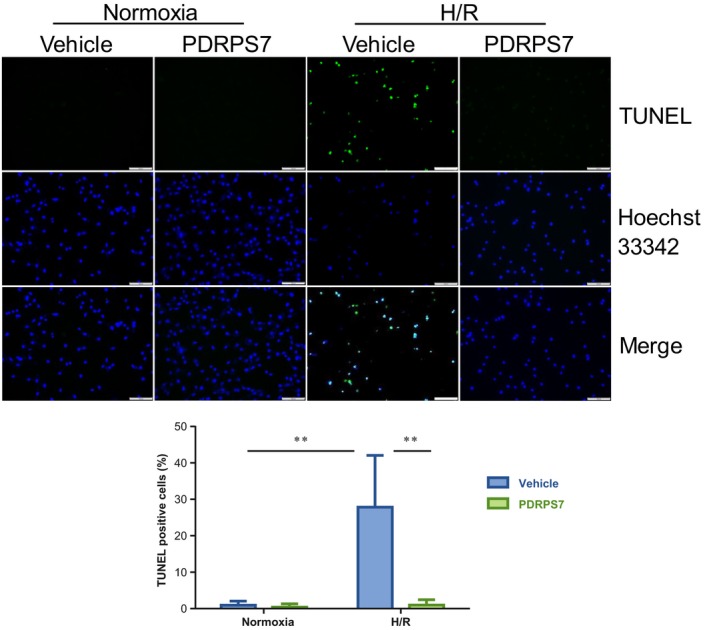
PDRPS7 decreased apoptosis following H/R. H9c2 cells were treated with PDRPS7 1 hr prior to H/R. TUNEL assay was performed to examine cell apoptosis (green). Hoechst 33342 was used to counterstain nuclei (blue). The staining was examined by a fluorescence microscopy at a magnification of 200×. The percentage of apoptotic cells over total cells was calculated. Scale bar = 100 μm. Data were shown as mean ± SD and analyzed using two‐way ANOVA followed by *post hoc* test. **P < 0.01, *n* = 11/group.

To further confirm the cell protective effects of PDRPS7 in H/R‐induced injury, we performed TUNEL assay to examine cell apoptosis. As shown in Fig. 6, H/R challenge significantly increased TUNEL‐positive cells by 3841.6% compared with that in vehicle‐treated normoxia controls (*P < *0.01). However, PDRPS7 pretreatment significantly inhibited H/R‐induced increase in TUNEL‐positive cells by 97.5% compared with the vehicle‐treated H/R group (*P < *0.01). PDRPS7 administration did not induce apoptosis in the normoxia + PDRPS7 group. Besides, scrambled peptide pretreatment showed no effect on H/R‐induced increase in TUNEL‐positive cells, compared with that in the vehicle‐treated H/R group (Fig. S1A).

### PDRPS7 reduces intracellular ROS generation following H/R

**Fig. 7:**
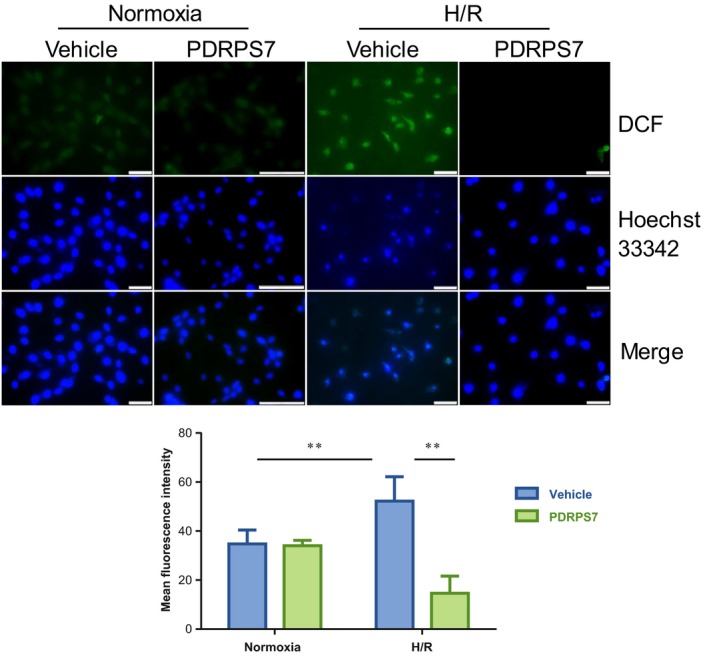
PDRPS7 decreased intracellular ROS content following H/R. H9c2 cells were treated with PDRPS7 1 hr prior to H/R. ROS content was examined by DCFH‐DA assay. The staining was examined by a fluorescence microscopy at a magnification of 400×. The percentage of apoptotic cells over total cells was calculated. Scale bar = 50 μm. Data were shown as mean ± SD and analyzed using two‐way ANOVA followed by *post hoc* test. **P < 0.01, *n* = 11/group.

Oxidative stress is one of the prominent mediators in I/R‐induced cardiac injury 24. To examine whether ROS was involved in protection by PDRPS7, we examined intracellular ROS content using DCFH‐DA assay. Cells were pretreated with PDRPS7 1 hr prior to H/R stimulation. H/R significantly increased ROS content in H9c2 cells by 46.1%, compared with vehicle‐treated normoxia controls (Fig. 7, *P < *0.01). However, the H/R‐increased ROS generation was attenuated (71.3%) with PDRPS7 pretreatment, when compared with the vehicle‐treated H/R group (*P < *0.01). Significantly, scrambled peptide pretreatment did not reduce the H/R‐induced ROS generation, compared with that in the vehicle‐treated H/R group. Instead, it raised the ROS levels (Fig. S1B, *P < *0.05).

### PDRPS7 does not improve H_2_O_2_‐treated cell survival

**Fig. 8:**
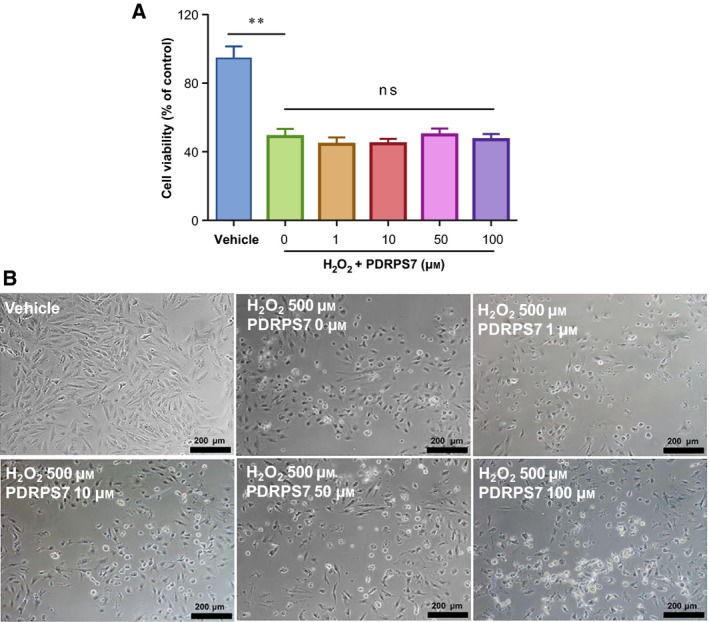
PDRPS7 did not impact cell injury following oxidant exposure. H9c2 cells were treated with PDRPS7 at the indicated concentrations 1 hr prior to H_2_O_2_ exposure. Cell injury was examined by MTT assay (A). Cell morphology was examined by phase‐contrast microscopy at a magnification of 100 × (B). Scale bar = 200 μm. Data were shown as mean ± SD and analyzed using one‐way ANOVA followed by *post hoc* test. *n* = 4/group.

The reduction in ROS content by PDRPS7 in H/R‐treated H9c2 cells motivated us to investigate whether PDRPS7 could attenuate the oxidant‐induced cell injury. To induce oxidative stress, H9c2 cells were treated with 500 μm H_2_O_2_ for 3 h. Both MTT and morphological analysis showed that H_2_O_2_ treatment (500 μm H_2_O_2_/0 μm PDRPS7) significantly reduced cell viability, respectively, when compared with the vehicle control (Fig. 8A,B). Unexpectedly, H_2_O_2_‐induced cell injury was not affected by pretreatment with PDRPS7 at concentrations of 0, 1, 10, 50, and 100 μm (Fig. 8A,B).

### PDRPS7 pretreatment attenuates H/R‐triggered cell apoptosis of H9c2 cells by inactivating JNKs

**Fig. 9:**
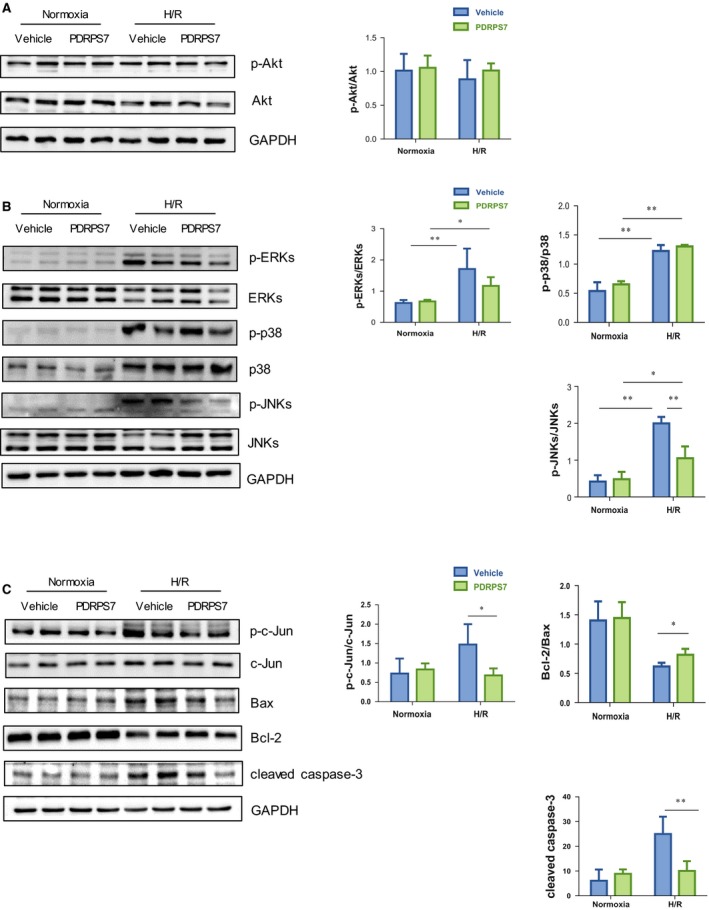
PDRPS7 pretreatment attenuated H/R‐triggered cell apoptosis of H9c2 cells by inactivating JNKs. H9c2 cells were treated with PDRPS7 1 hr prior to H/R. Cells were collected for immunoblotting analysis of phosphorylation of Akt (A); MAPKs (B); c‐Jun, p‐c‐Jun, Bax, Bcl‐2, and cleaved caspase‐3 (C). Data were shown as mean ± SD and analyzed using two‐way ANOVA followed by *post hoc* test. **P < 0.01 and *P < 0.05, *n* = 4–5/group.

We have previously shown that activation of Akt signaling plays roles in the protection against cardiac I/R injury 2. To investigate whether PDRPS7‐ induced protection involves Akt signaling, we examined Akt phosphorylation using immunoblotting analysis. However, no significant changes of Akt phosphorylation were observed among all the groups (Fig. 9A). MAPKs including ERKs, p38, and JNKs have been shown to play roles in mediating cardiac I/R injury 4, 11, 25. Immunoblotting analysis revealed that H/R increased phosphorylation levels of ERKs, p38 MAPK as well as JNKs in H9c2 cells, respectively, when compared with their vehicle‐treated normoxia controls (Fig. 9B, *P < *0.01). However, the H/R‐induced increases in ERKs and p38 MAPK phosphorylation were not changed by PDRPS7 pretreatment, compared with their vehicle‐treated H/R groups. Interestingly, PDRPS7 pretreatment significantly attenuated the H/R‐induced increase of JNKs phosphorylation levels when compared with the vehicle‐treated H/R group (*P < *0.01). We also examined the protein expression levels of c‐Jun, p‐c‐Jun, Bax, Bcl‐2 and cleaved caspase‐3, the downstream signaling effect of JNK related to cellular apoptosis. Figure 9C shows that 50 μm PDRPS7 significantly decreased the level of cleaved caspase‐3 (*P* < 0.01), the ratio of p‐c‐Jun/c‐Jun (*P* < 0.05), and increased the ratio of Bcl‐2/Bax (*P* < 0.05), respectively, compared with the vehicle‐treated H/R group. However, scrambled peptide pretreatment showed no effect on H/R‐induced increase in the level of cleaved caspase‐3 and decrease in the ratio of Bcl‐2/Bax, compared with the vehicle‐treated H/R group (Fig. S2).

### Activating JNKs diminishes the protection of PDRPS7 against H/R‐evoked cell injury

**Fig. 10:**
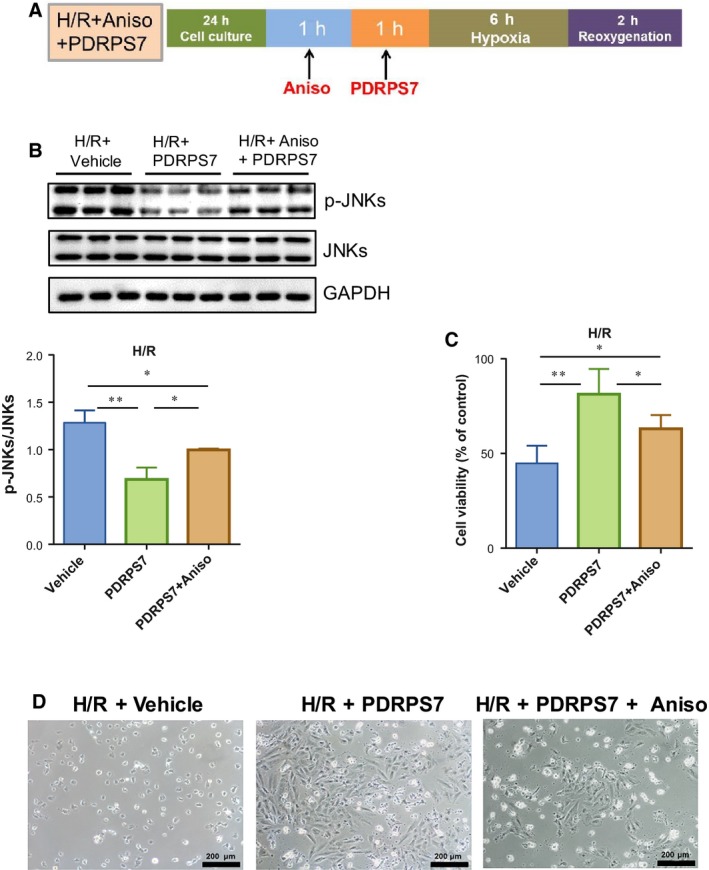
Activation of JNKs diminished cytoprotection of PDRPS7 against H/R. H9c2 cells were pretreated with Aniso for 1 hr followed by PDRPS7 administration. After PDRPS7 administration for 1 hr, cells were subjected to H/R. The treatment protocol (A). JNK activation was examined by immunoblotting analysis (B). Cell injury was evaluated by MTT assay (C) and morphological examination, scale bar = 200 μm (D). Data were shown as mean ± SD and analyzed using one‐way ANOVA followed by *post hoc* test. **P < 0.01 and *P < 0.05, *n* = 4–8/group.

To determine whether the inactivation of JNKs mediates the protection effects of PDRPS7, we employed Aniso, a widely used JNKs activator 26. The treatment protocol is shown in Fig. 10A. As expected, the PDRPS7‐induced decrease in JNKs phosphorylation was reversed by Aniso treatment in H/R + PDRPS7 + Aniso group, compared with the H/R + PDRPS7 group (Fig. 10B, *P < *0.05). Notably, MTT assay showed that Aniso administration diminished the PDRPS7‐induced survival protection against H/R stimulation compared with H/R + PDRPS7 group (Fig. 10C, *P < *0.05). In support of this finding, morphological examination demonstrated that the PDRPS7‐induced cell protection against H/R stimulation was partially diminished by Aniso (Fig. 10D).

### PDRPS7 attenuates H/R‐induced NRCMs injury

**Fig. 11:**
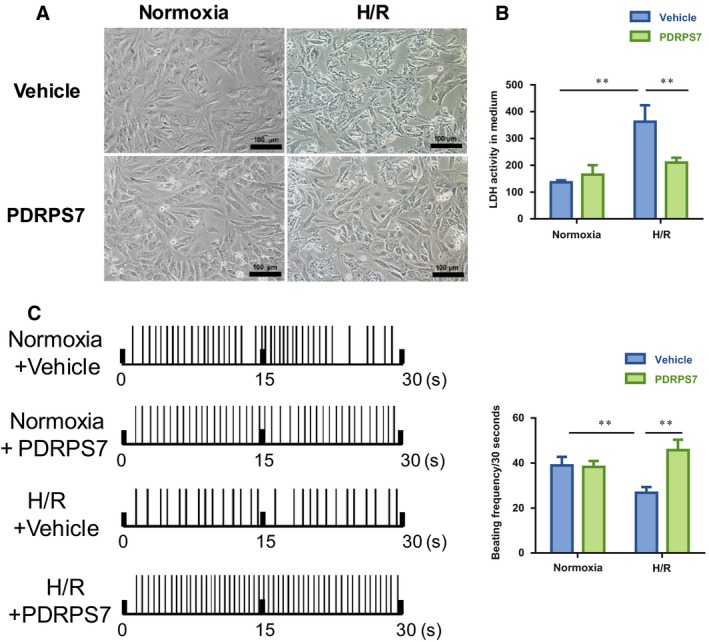
PDRPS7 attenuated H/R‐induced NRCMs injury. NRCMs were pretreated with PDRPS7 1 hr prior to H/R. Cell morphology was examined by phase‐contrast microscopy at a magnification of 200×. Scale bar = 100 μm, *n* = 4/group (A). LDH leakage (B) and the beating frequency (C) were tested. Data were shown as mean ± SD and analyzed using two‐way ANOVA followed by *post hoc* test. **P < 0.01, *n* = 4/group.

To detect whether the peptide PDRPS7 plays a role in H/R‐induced injury in NRCMs, we pretreated NRCMs with PDRPS7 (50 μm). As Fig. 11A shows, the H/R+ vehicle group showed cell shrinkage and cell fragmentation with inconspicuous edges, and the morphological abnormalities were rescued by peptide PDRPS7. Additionally, LDH activity assay demonstrated that the peptide PDRPS7 decreased the H/R‐induced LDH activity in NRCMs (Fig. 11B, *P < *0.01). The beating frequency decreased in the H/R+ vehicle group, compared with that in the normoxia + vehicle group. The peptide PDRPS7 pretreatment could significantly increase the beating frequency (Fig. 11C, *P < *0.01).

## Discussion

The significant finding of this study is that PDRPS7 attenuated H/R‐induced H9c2 cells and NRCMs injury. We also noticed that the H/R‐induced JNKs activation was suppressed by PDRPS7. However, pharmacological activation of JNKs diminished the PDRPS7‐induced cellular protection. Taken together, the data suggest that PDRPS7 exerts cardioprotective effects against stimulated I/R injury through inactivation of JNKs.

By using nanoflow liquid chromatography coupled online with an LTQ‐Orbitrap Velos mass spectrometer to compare peptidomic profiling changes induced by acute ischemic–hypoxia in primary cultured neonatal rat myocardial cells, we recently identified 220 differentially expressed peptides originating from 119 proteins. Among them, 37 peptides were upregulated and 183 were downregulated in cardiomyocytes exposed to hypoxia/ischemia conditions 18. To investigate their biological roles in ischemic hearts, we selected one peptide, PDRPS7, from the 37 upregulated peptides and examined the role in H/R injury in rat cardiomyoblast H9c2 cells and NRCMs. We found that peptide PDRPS7 with the amino acid sequence of TGKDVNFEFPEFQL significantly improved cell survival and attenuated apoptosis in H9c2 cells following H/R. We also examined the effect of PDRPS7 administration on H/R‐induced NRCMs injury. PDRPS7 significantly decreased the level of medium LDH and improved the beating rate in H/R‐treated NRCMs, which providing evidences for the functional data. Furthermore, we also conducted the experiments that treating the H9c2 cells with PDRPS7 for 4 h after the 6 h hypoxia, showing that PDRPS7 also has an effect on alleviating cell abnormal morphology and cell injury (Fig. S3). The data suggest the cardioprotective effects of the novel peptide PDRPS7 identified *in vitro*.

PI3K/Akt signaling and MAPK signaling have been shown to play important roles in the pathogenesis of cardiac I/R injury. Of particular interest, we demonstrated recently that Akt mediates the HSPA12B‐induced cardioprotection against I/R‐induced injury in mice 2. However, we found no effects of PDRPS7 peptide on Akt phosphorylation in H/R‐exposed H9c2 cells, suggesting that the protective effect of PDRPS7 was not mediated by Akt‐dependent signaling. Interestingly, in this study, we noticed that I/R‐induced activation of JNKs was suppressed by PDRPS7 pretreatment. I/R‐induced activation of ERKs and p38 was not affected by PDRPS7. These findings suggest that JNK inactivation may be involved in the cellular protection of PDRPS7. JNKs belong to a family of MAPKs, which are activated in response to various stress stimuli such as ultraviolet radiation, oxidative stress, heat and osmotic shock, and cardiac I/R injury 9. In addition, JNK signaling pathway has been shown to play critical roles in regulating cell fate, being implicated in a multitude of diseases ranging from cancer to ischemic immunological/inflammatory conditions 9, 27. JNKs cause changes to gene transcription, resulting in biological responses such as inflammation and/or apoptosis 9, 28. Many effects of JNKs are mediated by transcription factors of the activator protein‐1 family, of which c‐Jun is the most commonly known. c‐Jun and JNKs pathway not only mediate apoptosis but also the cell cycle arrest 29. In the downstream cascade of apoptotic signaling pathways, the release of proteins of the Bcl‐2 family that mediate mitochondrial permeability can affect apoptosis. The balance between pro‐apoptotic proteins (e.g., Bax, Bad, and Bid) and anti‐apoptotic proteins (Bcl‐2, Bcl‐W, Bfl‐1) is the key to cytochrome C release, which forms the apoptosome (with Apf‐1 and caspase‐9), that cleaves and thereby activates caspase‐3 30. Our present study demonstrates that PDRPS7 protects cardiomyocytes from H/R injury by inhibiting the activation of JNKs and c‐Jun, and altering the expression of related molecules in the downstream apoptotic signaling pathway, including increasing the Bcl‐2/Bax ratio and reducing cleaved caspase‐3 levels. Notably, we found that activation of JNKs diminished PDRPS7‐induced cellular protection in H9c2 cells following H/R. Taken together, our data indicate that PDRPS7 protects cardiomyocytes from H/R injury through, at least in part, the suppression of activation of JNKs.

During I/R, ROS is generated, causing activation of MAP kinases MLK3 and ASK1 which can phosphorylate MAP kinases MKK4/7, and, ultimately, JNK phosphorylation and activation. In this study, we found that H/R‐induced increases in intracellular ROS content were attenuated by PDRPS7 administration, suggesting that attenuation of oxidative stress may be involved in PDRPS7‐induced protection in H9c2 cells against H/R challenge. To clarify this hypothesis, we examined the effects of PDRPS7 on H_2_O_2_‐induced H9c2 cell injury. Unexpectedly, PDRPS7 showed no protective effects in cell injury of H_2_O_2_‐treated cells. The data suggest that the PDRPS7‐induced inactivation of JNKs in H/R‐exposed cardiac cells was ROS independent.

In summary, our study demonstrated that PDRPS7 protects cardiomyocytes from H/R‐induced cell death through, at least in part, inactivation of JNKs. The study identified PDRPS7 as a novel cardioprotective peptide in cardiac I/R injury.

## Conflicts of interest

The authors declare no conflict of interest.

## Author contributions

LC and YD conceived and designed the project, YD and SC acquired the data, SC and LJ analyzed and interpreted the data, and ZZ and YD wrote the paper.

## Supporting information


**Fig. S1.** Scrambled peptide pretreatment showed no effect on H/R‐induced cell apoptosis and increased ROS. H9c2 cells were treated with scrambled peptide 1 hr prior to H/R. TUNEL assay was performed to examine cell apoptosis (green) (A). Hoechst 33342 was used to counterstain nuclei (blue). The staining was examined by a fluorescence microscopy at a magnification of 200×. Scale bar = 100 μm. Data were shown as mean ± SD and analyzed using unpaired *t*‐test. ns, no significance, *n* = 3/group. ROS content was examined by DCFH‐DA assay (B). Hoechst 33342 was used to counterstain nuclei (blue). The staining was examined by a fluorescence microscopy at a magnification of 400×. The percentage of apoptotic cells over total cells was calculated. Scale bar = 50 μm. Data were shown as mean ± SD and analyzed using unpaired *t*‐test. *P < 0.05, *n* = 3/group.
**Fig. S2.** Scrambled peptide did not affect the expression of apoptosis‐related proteins. H9c2 cells were treated with scrambled peptide 1 hr prior to H/R. Cells were collected for immunoblotting analysis of Bcl‐2, Bax, cleaved caspase‐3. Data were shown as mean ± SD and analyzed using one‐way ANOVA followed by post‐hoc test. ns, no significance, *n* = 3/group.
**Fig. S3.** PDRPS7 post‐hypoxia treatment improved cell survival induced by H/R. H9c2 cells were exposed to hypoxia for 6 h, and then treated with PDRPS7 under reoxygenation for 4 h. The treatment protocol (A). Cell morphology was examined by phase‐contrast microscopy at a magnification of 200× (B), scale bar = 100 μm, *n* = 4/group. Cell injury was determined by MTT analysis (C) and LDH leakage (D). Data were shown as mean ± SD and analyzed using unpaired *t*‐test. **P < 0.01 and *P < 0.05; *n* = 4/group.
