# Supplementary figures and images for: PDRPS7 protects cardiac cells from hypoxia/reoxygenation injury through inactivation of JNKs
Source: FEBS Open Bio. 2020 Mar 16;10(4):593–606. doi: 10.1002/2211-5463.12822 (PMC7137793; doi:10.1002/2211-5463.12822)

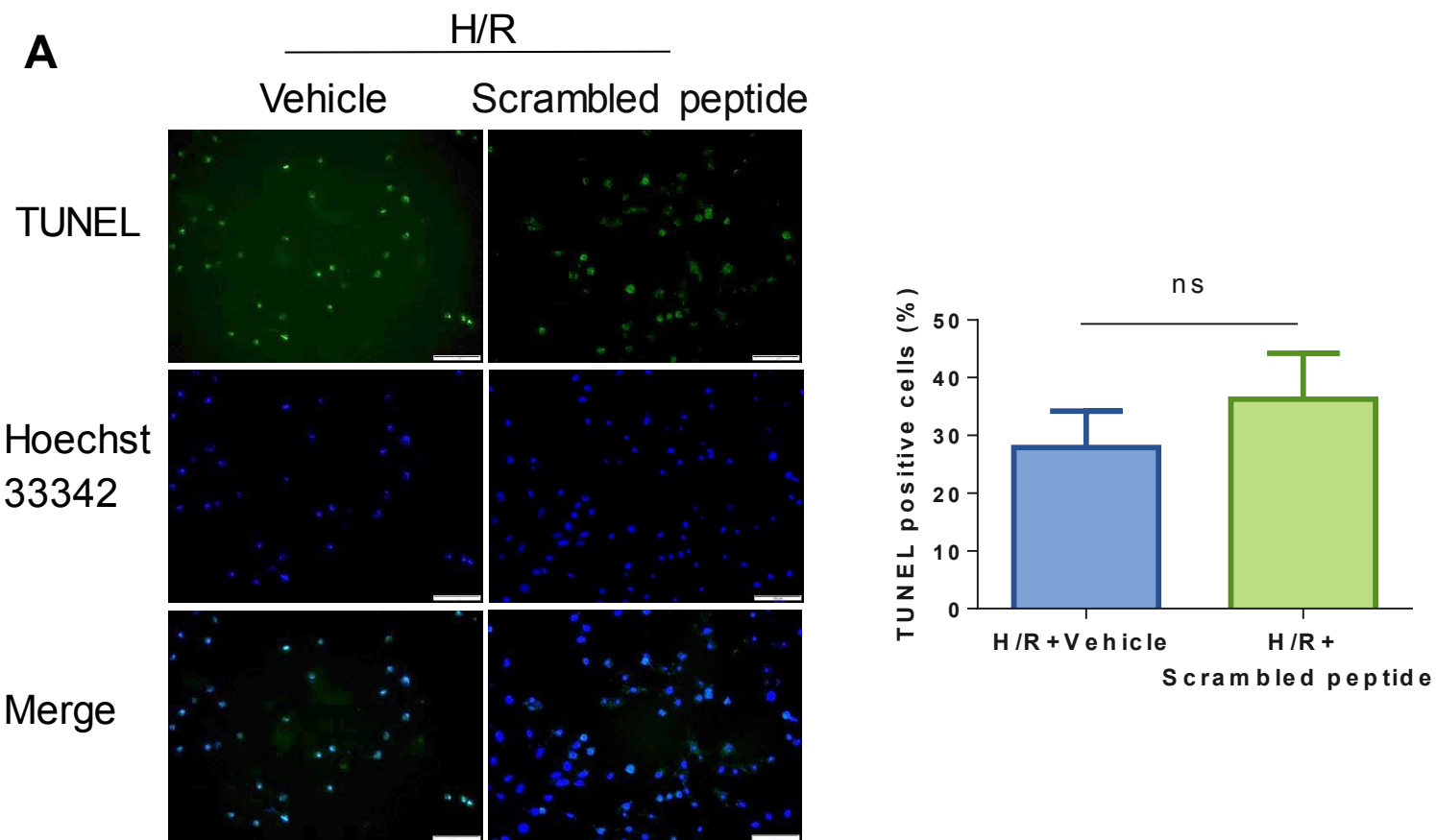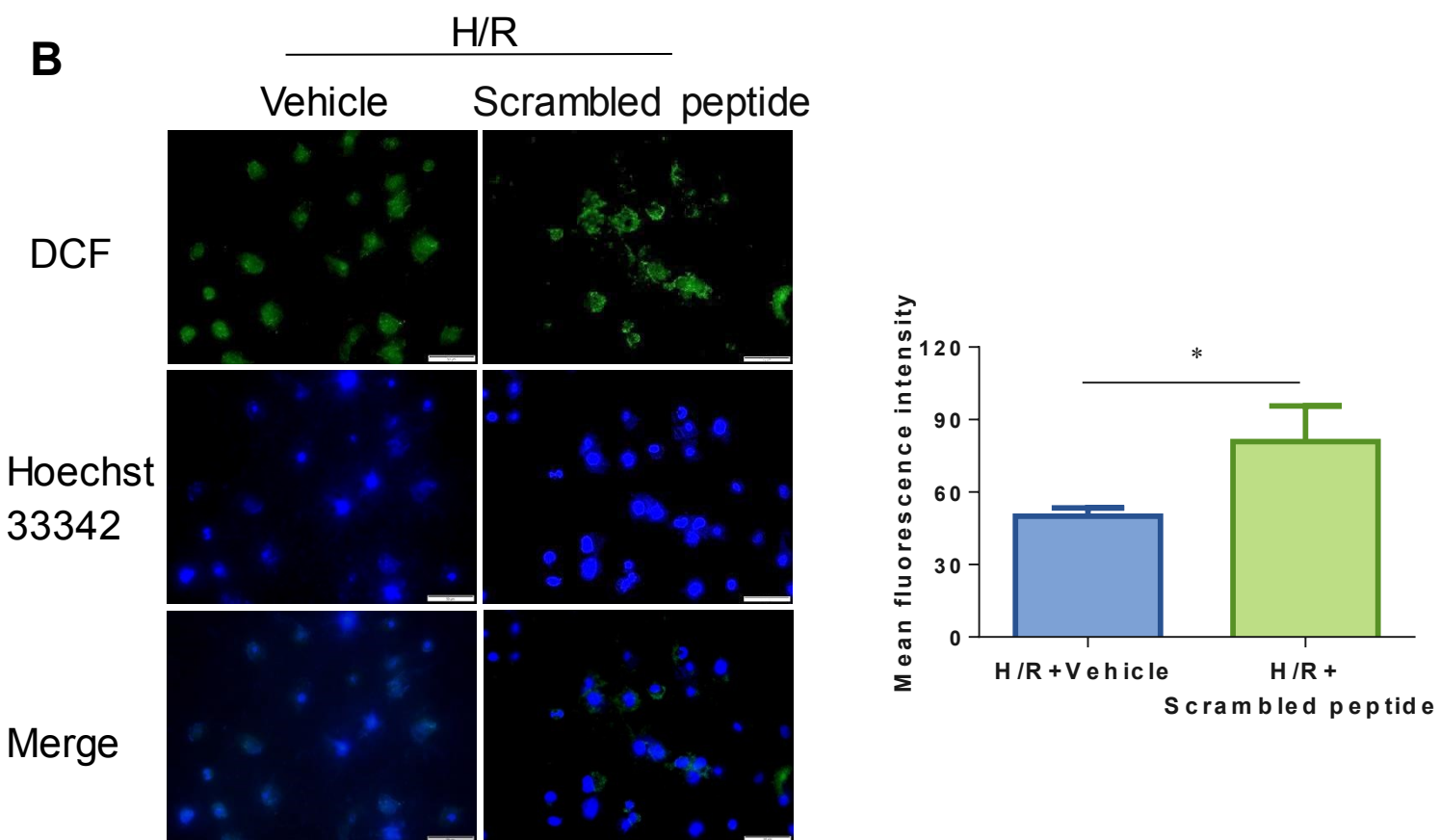

**Figure S1**

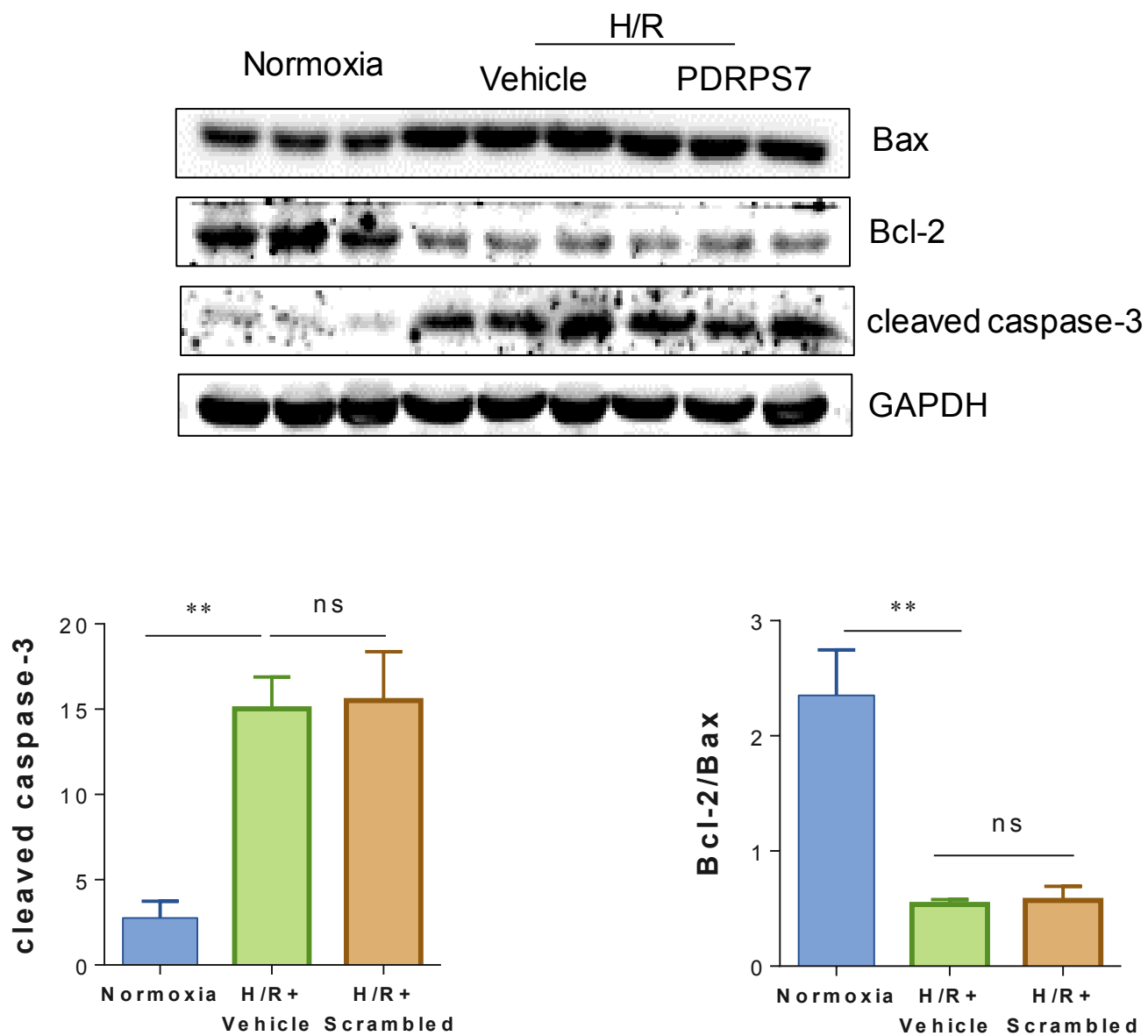

**Figure S2**

**A**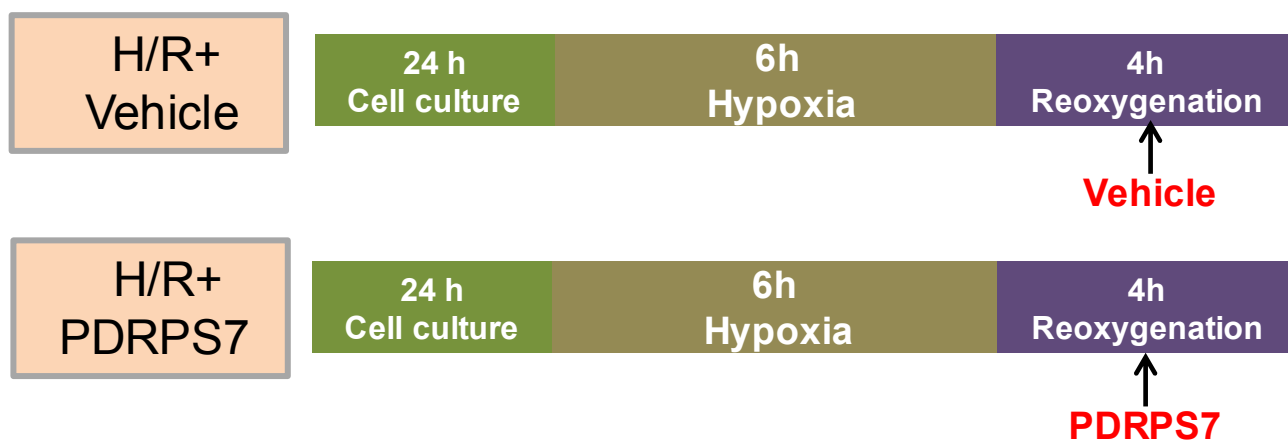**B**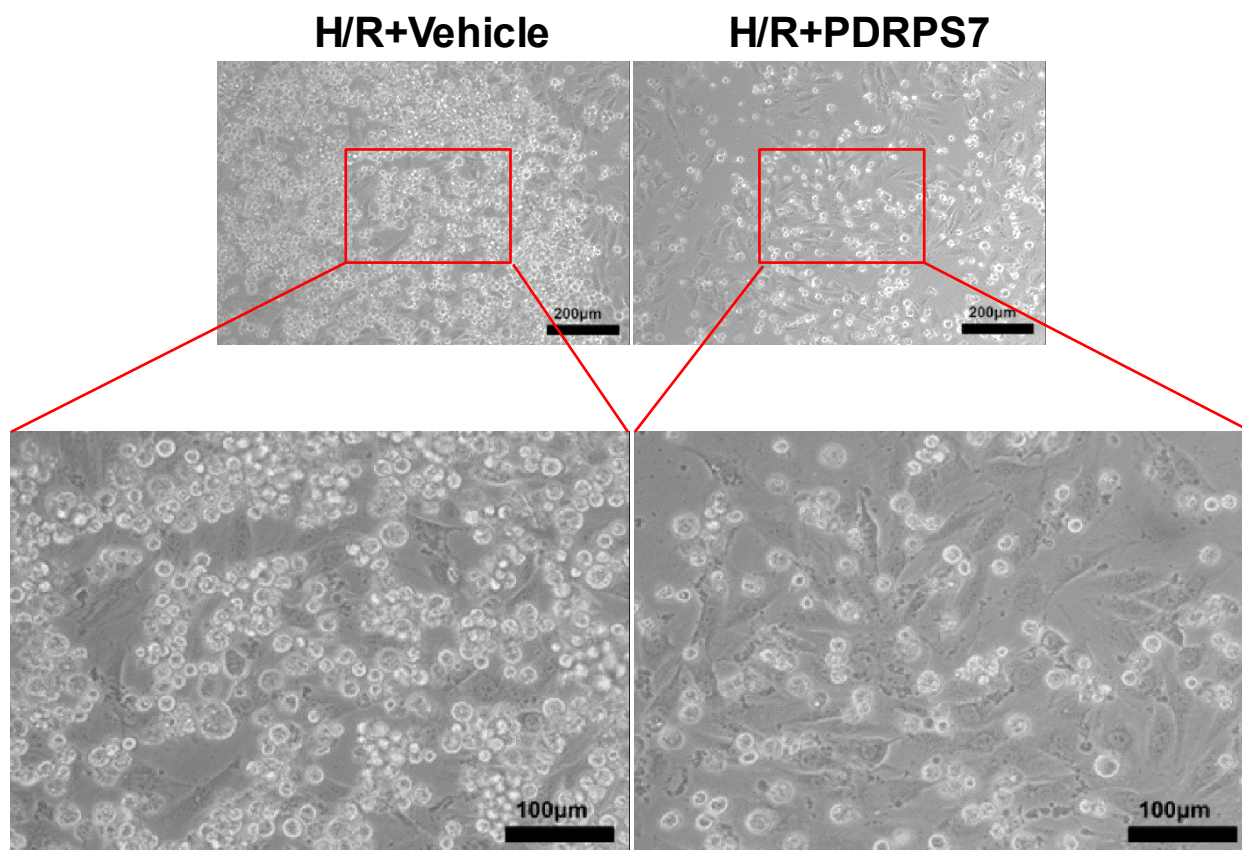**C**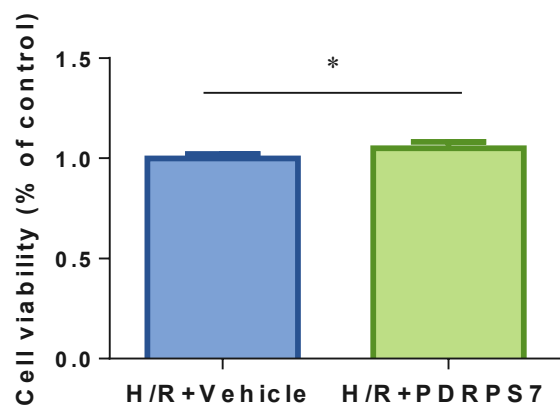**D**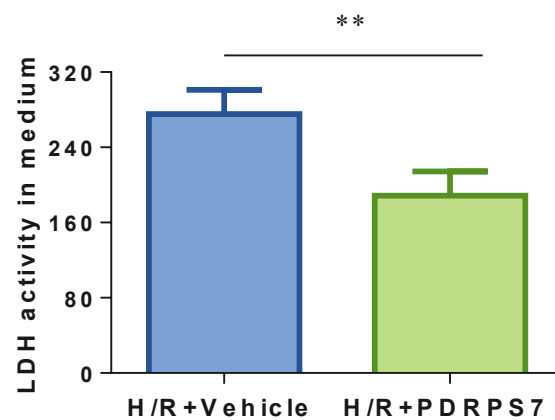**Figure S3**

Supplement: Supplementary file 1 — Fig. S1. Scrambled peptide pretreatment showed no effect on H/R‐induced cell apoptosis and increased ROS. H9c2 cells were treated with scrambled peptide 1 hr prior to H/R. TUNEL assay was performed to examine cell apoptosis (green) (A). Hoechst 33342 was used to counterstain nuclei (blue). The staining was examined by a fluorescence microscopy at a magnification of 200×. Scale bar = 100 μm. Data were shown as mean ± SD and analyzed using unpaired t‐test. ns, no significance, n = 3/group. ROS content was examined by DCFH‐DA assay (B). Hoechst 33342 was used to counterstain nuclei (blue). The staining was examined by a fluorescence microscopy at a magnification of 400×. The percentage of apoptotic cells over total cells was calculated. Scale bar = 50 μm. Data were shown as mean ± SD and analyzed using unpaired t‐test. *P < 0.05, n = 3/group. Fig. S2. Scrambled peptide did not affect the expression of apoptosis‐related proteins. H9c2 cells were treated with scrambled peptide 1 hr prior to H/R. Cells were collected for immunoblotting analysis of Bcl‐2, Bax, cleaved caspase‐3. Data were shown as mean ± SD and analyzed using one‐way ANOVA followed by post‐hoc test. ns, no significance, n = 3/group. Fig. S3. PDRPS7 post‐hypoxia treatment improved cell survival induced by H/R. H9c2 cells were exposed to hypoxia for 6 h, and then treated with PDRPS7 under reoxygenation for 4 h. The treatment protocol (A). Cell morphology was examined by phase‐contrast microscopy at a magnification of 200× (B), scale bar = 100 μm, n = 4/group. Cell injury was determined by MTT analysis (C) and LDH leakage (D). Data were shown as mean ± SD and analyzed using unpaired t‐test. **P < 0.01 and *P < 0.05; n = 4/group. [file FEB4-10-593-s001.pdf]
